# Supplementary material for: Molecular Evolutionary Analyses of the Pseudomonas-Derived Cephalosporinase Gene
Source: Microorganisms. 2023 Mar 1;11(3):635. doi: 10.3390/microorganisms11030635 (PMC10057138; doi:10.3390/microorganisms11030635)
Supplement: Supplementary file 1 [file microorganisms-11-00635-s001.zip › microorganisms-2072466-supplementary/supplementary_figure_table_revised_2/revised_supplementary_figure.pdf]

microorganisms

MDPI

Supplementary Figure

Cluster A	70	GLASKEDGRRVTPETLFEIGSVSKTFTATLAGYALTQDKMRLDDRA	115
Cluster B	70	GLASKEDGRRVTPETLFEIGSVSKTFTATLAGYALAQDKMRLDDRA	115
Cluster C	70	GLASKEDGRRVTPETLFEIGSVSKTFTVTLAGYALAQDKMRLDARA	115
Cluster D	70	GLASKEDGRRVTPETLFEIGSVSKTFTATLAGYALAQDKMRLDDRA	115
Cluster E	70	GLASKEDGRQVTPETLFEIGSVSKTFTATLAGYALAQDKMRLDDRA	115
Cluster F	70	GLASKEDGRRVTPETLFEIGSVSKTFTATLAGYALAQDKMRLDDRA	115
Cluster G	70	GLASKEDGRRVTPETLFEIGSVSKTFTATLAGYALAQDKMRLDDRA	115
Cluster H	70	GVASKEDARKVTPETLFEIGSVSKTFTATLAGYALAQDKMRLDDRA	115

Cluster A	160	DYYRQWQPTYAPGSQRLYSNPSIGLFGYLAARSLGQPFERLMEQQV	205
Cluster B	160	DYYRQWQPTYAPGSQRLYSNPSIGLFGYLAARSLGQPFERLMEQQV	205
Cluster C	160	DYYRQWQPTYAPGSQRLYSNPSIGLFGYLAARSLGQPFERLMEQQL	205
Cluster D	160	DYYRQWQPTYAPGSQRLYSNPSIGLFGYLAARSLGQPFERLMEQQL	205
Cluster E	160	DYYRQWQPTYAPGSQRLYSNPSIGLFGYLAARSLGQPFERLMEQQL	205
Cluster F	160	DYYRQWQPTYTPGSQRLYSNPSIGLFGYLAARSLGQPFERIMEQQL	205
Cluster G	160	DYYRQWQPTYAPGSQRLYSNPSIGLFGYLAARSLGQPFERIMEQRL	205
Cluster H	160	DYYRQWQPTYAPGSHRQYSNPSIGLFGYLAARSLGQPFERSMERQL	205

Cluster A	322	PHRIARLPAPQALEGQRLLNKTGSTNGFGAYVAFVPGRDLGLVILA	367
Cluster B	322	PHRIARLPAPQALEGQRLLNKTGSTNGFGAYVAFVPGRDLGLVILA	367
Cluster C	322	PHRIARLPAPQALEGQRLLNKTGSTNGFGAYVAFVPGRDLGLVILA	367
Cluster D	322	PHRIARLPAPQALEGQRLLNKTGSTNGFGAYVAFVPGRDLGLVILA	367
Cluster E	322	PHRIARLSAPQALEGQRLLNKTGSTNGFGAYVAFIPGRDLGLVILA	367
Cluster F	322	PHRIARLPAPQAPEGQRLLNKTGSTNGFGAYVAFIPGRDLGLVILA	367
Cluster G	322	PHRVARLPAPQALEGQRLLNKTGSTNGFGAYVAFIPGRDLGLVILA	367
Cluster H	322	AHKVARLPAPQALDGQRLLNKTGSTNGFGAYLAFIPGRDVGLVILA	367

Supplementary Figure S1. Multiple sequence alignment of representative *Pseudomonas*-derived cephalosporinase (PDC) amino acid sequences from clusters A to H. Each active site motif of PDC is highlighted in light yellow, light blue, and light green, respectively.
