# Supplementary material for: Molecular Evolutionary Analyses of the Pseudomonas-Derived Cephalosporinase Gene
Source: Microorganisms. 2023 Mar 1;11(3):635. doi: 10.3390/microorganisms11030635 (PMC10057138; doi:10.3390/microorganisms11030635)
Supplement: Supplementary file 1 [file microorganisms-11-00635-s001.zip › microorganisms-2072466-supplementary/supplementary_figure_table_revised_2/samples present study.pdf]

Supplementary Table S1. The genome samples used in the present study

Genebank accession No.	Country	Collection year
ALOF01000013	Australia	1975
AMVN01000116	Spain	2009
AOBK03000006	India	2012
AOIH01000020	Spain	2010
AXZJ01000019	India	2013
AYSK01000275	India	2012
AZBO01000022	India	2013
CP007399	USA	2012
CP010555	USA	1979
CP011369	Netherlands	2013
CP015001	Brazil	1997
CP020560	India	2014
CP026680	USA_ New York	2012
CP028331	France	2015
CP028368	France	2015
CP028959	China	2012
CP041354	Germany	2012
CP049161	Australia	2016
CP054472	Canada	2015
FM209186	England	1988
FR822745	Romania	2004
FR822746	Romania	2004
FR822747	Romania	2001
JAFBLI010000001	China	2018
JF326295	Canada	2010
JF326297	Canada	2009
JF326299	Canada	2008
JF326301	Canada	2007
JGZW01000193	Canada	1988
JGZX01000002	Canada	1986
JGZY01000016	Canada	1982
JGZZ01000001	Canada	1987
JHEU01000936	Kazakhstan	2009

JJOD01000066	India	2004
JMBR02000059	India	1995
JMBS02000027	India	1996
JPFF01000002	USA	2012
JPYP01000015	USA	2008
JRGP01000011	United Kingdom	2009
KX689751	Viet Nam	2015
LBIH01000023	USA	2010
LFDG01000016	India	2013
LFDH01000041	India	2013
LJJO01000079	Australia	2012
LKAF01000012	United Kingdom	2015
LKPU01000016	Canada	2012
LLKP01000044	Canada	1966
LN831024	Czech Republic	1963
LNDM01000012	Germany	1995
LOHH01000034	USA	2007
LOHI01000014	USA	2008
LOHK01000021	USA	2006
LRSF01000036	USA	1999
LRSG01000027	USA	2004
LRSJ01000025	USA	2012
LRYI01000002	USA	2000
LRYK01000057	USA	2005
LRYN01000031	USA	2010
LRYO01000032	USA	2004
LRYR01000078	USA	2003
LRYU01000011	USA	2004
LRYW01000009	USA	2004
LRZF01000006	USA	2008
LRZJ01000022	USA	2005
LRZM01000007	USA	2006
LRZP01000006	USA	2012
LRZQ01000025	USA	2005
LRZU01000019	USA	2006

LSAA01000015	USA	2009
MF098272	USA	2016
MF098274	Canada	2016
MF098276	Canada	2016
MF098282	USA	2016
MF098283	USA	2016
MF098285	Taiwan	2016
MF098288	Australia	2016
MF098289	Japan	2016
MF098296	Canada	2016
MF098302	Japan	2016
MF098303	USA	2016
MF098305	United Kingdom	2016
MF281077	Spain	2016
MF281082	Spain	2016
NG_049892	Canada	2009
NG_049897	Canada	2007
NG_049899	Canada	2007
NG_049900	Canada	2009
NG_054965	Venezuela	2016
NG_054967	USA	2016
NG_054969	Canada	2016
NG_054971	USA	2016
NG_054973	USA	2016
NG_054974	Philippines	2016
NG_054975	Argentina	2016
NG_054978	Taiwan	2016
NG_054980	Brazil	2016
NG_054981	Panama	2016
NG_054984	Puerto Rico	2016
NG_054986	South Africa	2016
NG_054987	Colombia	2016
NG_054988	Colombia	2016
NG_054990	Canada	2016
NG_054991	Canada	2016

NG_054992	Canada	2016
NG_054993	USA	2016
NG_055274	Israel	2016
NG_055276	Morocco	2016
NG_055277	Tunisia	2016
NG_055278	United Kingdom	2016
NG_055279	Italy	2016
NG_055281	Saudi Arabia	2016
NG_055282	Portugal	2016
NZ_CP024630	China	2010
NZ_CP025049	USA	2014
NZ_CP053117	Australia	2017
NZ_CP053706	Taiwan	2003
NZ_CP053917	South Korea	2019
NZ_JABGNI010000019	United Kingdom	2015
NZ_JABGNL010000018	United Kingdom	2014
NZ_JABGNM010000019	United Kingdom	2014
NZ_JABGNR010000016	United Kingdom	2012
NZ_JABGNS010000015	United Kingdom	2012
NZ_JABGNU010000018	United Kingdom	2012
NZ_JABGOD010000016	United Kingdom	2017
NZ_JABGOH010000016	United Kingdom	2015
NZ_JABLSN010000010	Taiwan	2004
NZ_JACEKE010000029	United Kingdom	2013
NZ_JACEMH010000032	United Kingdom	2013
NZ_JACEPL010000013	France	2004
NZ_JACEPP010000010	France	2004
NZ_JACWHC010000005	Lebanon	2018
NZ_JAGFEQ010000011	Nigeria	2018
NZ_JAGMWR010000017	France	2018
NZ_JAHBSB010000003	USA	2021
NZ_JAHBSD010000008	USA	2020
NZ_JAHBSE010000010	USA	2020
NZ_JWJU01000006	Malaysia	2014
NZ_KI519031	USA	2013

NZ_LDKQ01000006	Canada	2011
NZ_LLLT01000002	France	1991
NZ_LLLU01000013	France	1992
NZ_LLMW01000047	USA	1995
NZ_LLOY01000001	France	1994
NZ_LLPR01000004	USA	2005
NZ_LLVD01000038	Tunisia	1998
NZ_LR130528	Switzerland	2017
NZ_LRSF01000036	USA	1999
NZ_LRSI01000037	USA	2011
NZ_LRZZ01000005	USA	2009
NZ_MVGX01000011	USA	1996
NZ_MWLA01000064	Spain	2005
NZ_MWWL01000204	Australia	2008
NZ_MWYK01000002	Canada	1988
NZ_MWZF01000043	Australia	2008
NZ_NION01000023	USA	2008
NZ_NMRS01000019	Pacific Ocean	2004
NZ_NSRU01000013	Australia	2007
NZ_NSTL01000015	Portugal	2005
NZ_NSTR01000013	USA	2005
NZ_NSWW01000015	Australia	2002
NZ_NSWZ01000003	Australia	2006
NZ_NSYH01000036	Australia	2008
NZ_PDLR01000002	USA	2001
NZ_QVCW02000019	USA	2001
NZ_QZXE01000193	Estonia	2013
NZ_QZYT01000074	Estonia	2012
NZ_RAEF01000002	Estonia	2012
NZ_RAFH01000060	Estonia	2005
NZ_RHSA01000022	Pakistan	2016
NZ_RHTA01000011	Pakistan	2016
NZ_RHTB01000087	Pakistan	2016
NZ_RRBJ01000023	United Kingdom	2016
NZ_RRBQ01000016	United Kingdom	2016

NZ_RRBR01000037	United Kingdom	2016
NZ_RRBV01000009	United Kingdom	2016
NZ_RRBX01000028	United Kingdom	2017
NZ_RRBY01000013	United Kingdom	2017
NZ_RWLY01000006	Canada	2014
NZ_RWOB01000002	Canada	2014
NZ_RWOI01000016	Canada	2014
NZ_RWOK01000007	Canada	2014
NZ_RWTN01000011	Canada	2007
NZ_RWUV01000016	Canada	2012
NZ_RWZL01000003	USA	2012
NZ_RXFD01000011	Australia	2009
NZ_RXFE01000007	Australia	2009
NZ_RXUS01000020	USA	2011
NZ_RXVS01000089	USA	2013
NZ_SDVP01000023	Mexico	2015
NZ_VZIY01000029	USA	2019
NZ_WOAW01000013	Australia	2005
NZ_WUTP01000019	Brazil	2017
NZ_WXEV01000083	India	2017
PQBU01000013	India	2009
PQGF01000020	Brazil	2009
PUEW01000019	Australia	2010
PVXJ01000009	France	2010
QBEA01000013	Brazil	1995
QBEB01000012	Brazil	1998
QBED01000005	Brazil	1995
QBEE01000019	Brazil	1995
QBEG01000009	Brazil	1994
QBEO01000005	Brazil	2015
QBES01000012	Brazil	2012
QDGE01000015	Australia	2003
QDGF01000032	Australia	2003
QDGJ01000026	Australia	2006
QDGM01000012	India	2004

QDGR01000016	Australia	1992
QDGS01000010	Australia	2003
QDGU01000015	Australia	2004
QDGX01000010	India	1997
QGSM01000012	Germany	2014
QJGW01000017	USA	2010
QJGX01000018	USA	2010
